# Pediatric Otolaryngology Telehealth in Response to COVID-19 Pandemic: Lessons Learned and Impact on the Future Management of Pediatric Patients

**DOI:** 10.1177/0003489420976163

**Published:** 2020-11-26

**Authors:** Ryan H. Belcher, James Phillips, Frank Virgin, Jay Werkhaven, Amy Whigham, Lyndy Wilcox, Christopher T. Wootten

**Affiliations:** 1 Pediatric Otolaryngology – Head and Neck Surgery at Vanderbilt Children’s Hospital, Nashville, TN, USA

**Keywords:** telehealth, telemedicine, pediatric otolaryngology, teleotoscopy, COVID-19, pandemic, survey, general pediatric otolaryngology, novel techniques in pediatric otolaryngology

## Abstract

**Background:**

Since the start of the COVID-19 pandemic outpatient medicine has drastically been altered how it is delivered. This time period likely represents the largest volume of telehealth visits in the United States health care history. Telehealth presents unique challenges within each subspecialty, and pediatric otolaryngology is no different. This retrospective review was designed to evaluate our division of pediatric otolaryngology’s experience with telehealth during the COVID19 pandemic.

**Methods:**

This study was approved by the Institutional Review Board at Vanderbilt University Medical Center. All telehealth and face-to-face visits for the month of April 2020 completed by the Pediatric Otolaryngology Division were reviewed. A survey, utilizing both open-ended questions and Likert scaled questions was distributed to the 16 pediatric otolaryngology providers in our group to reflect their experience with telehealth during the 1-month study period.

**Results:**

In April, 2020 our outpatient clinic performed a total of 877 clinic visits compared to 2260 clinic visits in April 2019. A total of 769 (88%) were telehealth visits. Telemedicine with video comprised 523 (68%) and telephone only comprised 246 (32%). There were 0 telehealth visits in April 2019. Interpretive services were required in 9.3% (N = 211) clinic visits in April 2019 and 7.5% (N = 66) of clinic visits in April 2020. The survey demonstrated a significant difference (*P* < .00002) in provider’s anticipated telehealth experience (mean 3.94, 95% CI [3.0632, 4.8118] compared to their actual experience after the study period (mean 7.5, 95% CI [7.113, 7.887].

**Conclusions:**

Despite low initial expectations for telehealth, the majority of our providers felt after 1 month of use that telehealth would continue to be a valuable platform post-pandemic clinical practice. Limited physical exam, particularly otoscopy, nasal endoscopy, and nasolaryngoscopy present challenges. However, with adequate information and preparation for the parents and for the physician some of the obstacles can be overcome.

## Introduction

The COVID-19 pandemic has drastically changed the landscape of healthcare delivery throughout the entire United States (U.S.) since its onset and potentially for the foreseeable future. “Shelter in place” orders from local governments and restriction on elective surgical cases, resulted in a dramatic decrease in face-to-face outpatient encounters for physicians and patients, particularly in the months of March and April, 2020. In order to lessen the financial and clinical impact of these decreased encounters, telehealth emerged as a viable outlet to provide needed care to patients.

Previously, a temporary and rapid pivot to telehealth has been shown to be useful and efficient for delivery of health care for pediatric patients in disaster situations.^1,2^ Murren-Boezem et al.^2^ reviewed their telemedicine visits with pediatric patients in 2017 when Hurricane Irma made landfall on the coast of Florida. This natural disaster left 60% of the state without electricity and disrupted healthcare facilities statewide. Even though wait times were increased for families the patient and provider satisfaction remained high. Telemedicine has also been successfully deployed to avert a cholera epidemic in Makakumbh Mela, India during a large gathering of people.^3,4^ While neither this environmental disaster or epidemiologic outbreak was on the scale of the current COVID-19 pandemic, their insights are still useful in how telehealth can be utilized during a healthcare emergency.

Very little is published on the unique telehealth experiences in pediatric otolaryngology and the inherent challenges faced when the physical exam is extremely limited. This retrospective and survey study was designed to evaluate our Pediatric Otolaryngology Division’s experience with telehealth during the COVID-19 pandemic to garner insights and offer lessons learned.

## Methods

This study was approved by the institutional review board at Vanderbilt University Medical Center. The terms “telehealth” and “telemedicine” are often used interchangeably, but the definitions differ between the two. Telehealth is a much broader term for electronic health encounters and for the purposes of this article, it will be used to encompass both telemedicine with video chat and telephone only encounters. Both telemedicine with video and telephone encounters are considered to be separate types of telehealth services. At our institution, in order to be scheduled for a telemedicine visit in our electronic medical record (EMR) platform the patient or their caregiver must register through a proprietary platform “My Health at Vanderbilt” (MHAV) at least 24 hours in advance of their scheduled visit. At least 1 caregiver per patient is registered in the EMR with their phone number and e-mail on file. Our clinic staff and nursing team would reach out to the families ahead of their appointment via phone call and email for instructions on how to set this platform up. MHAV is a free, Health Insurance Portability and Accountability Act (HIPAA) compliant online portal that enables patients to view their medical records, review test results, schedule appointments, and message their provider. If the patient has not properly signed up for MHAV at least 24 hours in advance of their visit, the telehealth encounter has to be scheduled as a telephone visit and cannot be billed as telemedicine with video encounter. The default HIPAA compliant video teleconferencing integrated with our EMR is Zoom (San Jose, CA).

We retrospectively reviewed all telehealth encounters completed by our tertiary pediatric otolaryngology practice in April 2020, which included telemedicine with video chat and telephone only appointments. Our Pediatric Otolaryngology Division at Vanderbilt Children’s Hospital is comprised of 7 pediatric otolaryngology fellowship trained physicians, 8 nurse practitioners (NP), and 1 physician’s assistant (PA). We also retrospectively compiled the number of telehealth visits within our division of pediatric otolaryngology for the past 13 months, the number of face-to-face visits for the month of April 2020, and the total number of outpatient clinic patients seen by our clinic faculty in April 2019. We chose to retrospectively review 13 months of telehealth visits so that it would include the time period from April 2019 through April 2020.

The compiled list of outpatient visits was extracted from our EMR system and downloaded securely into Excel (Microsoft, Redmond, Washington). Inclusion criteria included all completed telehealth visits for the month of April, 2020 and all previous months over the past 13 months. Completed face-to-face visits were also included from April 2020 and all clinic visits (face-to-face and telehealth) from April 2019. Exclusion criteria included patients that did not answer their phone or connect to the telemedicine video chat for their scheduled visit, and patients that did not show for their face-to-face appointment. If a caregiver successfully used the videoconferencing platform, but the patient was not present or not examined then this was billed as a telephone encounter instead of the telemedicine with video encounter.

A 19-question survey (non-validated) was sent to all pediatric otolaryngology providers that provided clinical care during April 2020 via email in May 2020. The survey was designed to evaluate the provider’s experiences over the previous month, challenges, and advice for future pediatric otolaryngology telehealth encounters. The survey had a mix of yes/no, multiple choice, Likert scale, and open-ended qualitative questions. All 16 surveys were returned within a week. Data from the surveys was downloaded in Excel. Tableau (Salesforce, Seattle, WA) software was used to create geographic information system mapping. Statistical analysis was performed using Excel and Wilcoxon-Mann-Whitney *U* test to compare differences.

## Results

Overall in the month of April 2020 our group completed 877 clinic visits. Of these 769 (88%) were telehealth visits and 108 (12%) were face-to-face clinic visits. The demographics of these visits are depicted in Table 1. Of the telehealth visits, 246 (32%) were conducted using telephone only and 523 (68%) were telemedicine with video. There were 374 females (42.6%) and 503 males (57.4%) in all clinic visits. The majority were English speaking patients (n = 811, 92.5%) with 66 families (7.5%) requiring an interpreter through the telehealth service for their visit. Interpretive services provided included Arabic, Chinese, Kirundi, Zomi, Portuguese, and Spanish language. Interpretation for Spanish language comprised 51/66 (77.3%) of the telehealth patients. All 57 encounters requiring interpretive services had their telehealth visit conducted utilizing telephone service only. Tennessee residents comprised 712 (93%) of the patients, 42 patients were from Kentucky, 11 from Alabama, 2 from Virginia, 1 from Mississippi, and 1 from Michigan.

**Table 1.** Demographics of Outpatient Clinic Visits from April 2019 and April 2020.

Vanderbilt Pediatric Otolaryngology Division		April 2019	April 2020
Number of outpatient clinic visits		2069	877
	Face-to-face	2069	108
	Telemedicine with video	0	523
	Telephone only	0	246
Female		44% (N = 994)	42.6% (N = 374)
Male		56% (N = 1266)	57.4% (N = 503)
Interpreting services needed		9.3% (N = 211)	7.5% (N = 66)
States represented		16	6

**Figure 1.**
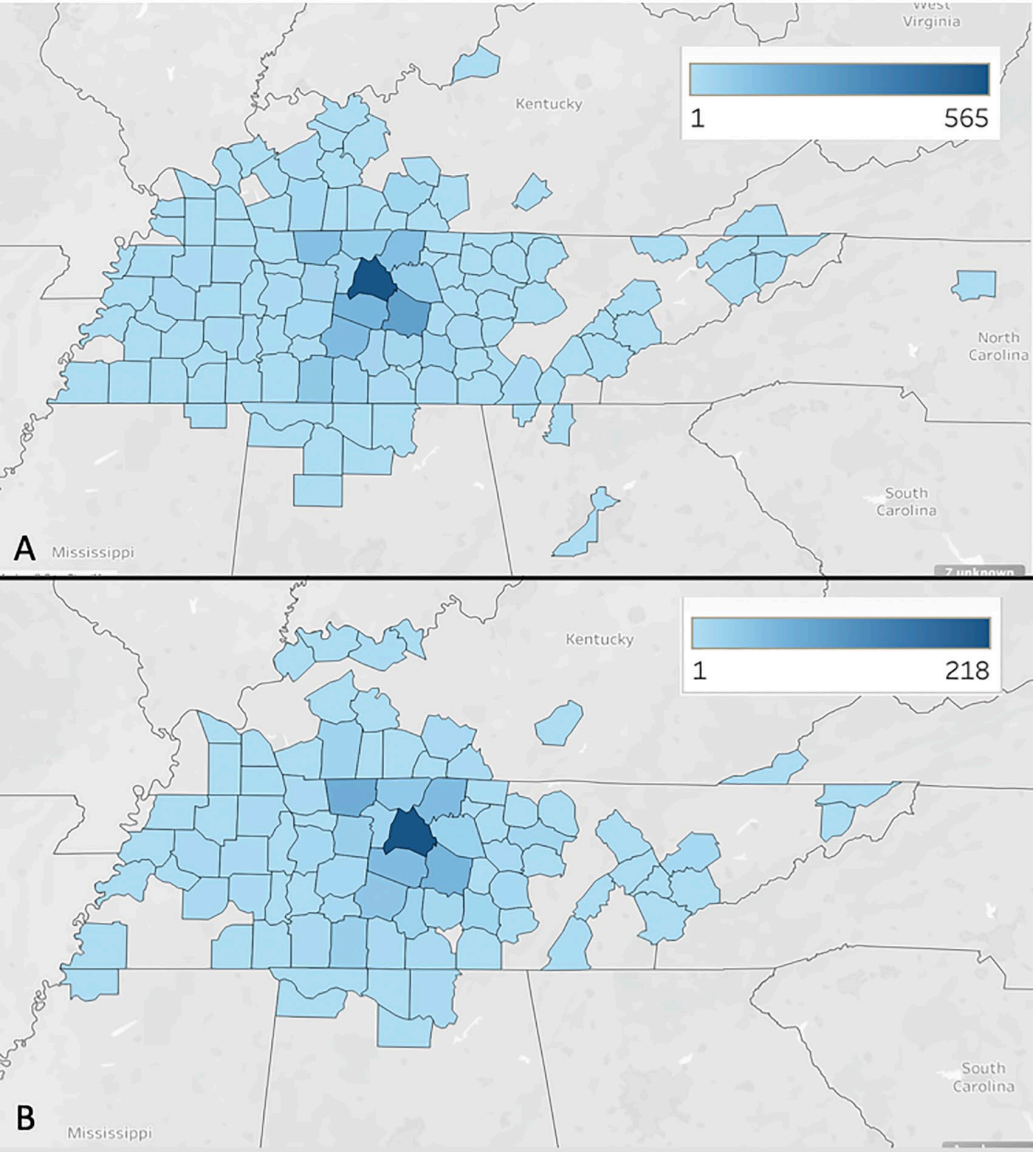
(A) This represents the number of outpatient visits in April 2019, by county of origin, which were all face-to-face encounters. The more dense/darker the county, the more outpatient clinic visits from that county. (B) This represents number of total outpatient visits per county in April 2020. The total number of clinic visits is lower than April 2019, but the geographical distribution of patient populations is similar.

In April 2019 there were 0 telehealth visits and all 2260 visits were performed face-to-face. There were 994 females (44%) and 1266 males (56%) in this cohort of patients. Interpreters were needed in 211 total patients (9.3%) with Spanish language representing the large majority of those requiring an interpreter with 155 (73%) clinic visits. There were 16 different states represented with outpatient visits with patients from Tennessee representing 2099 (93%) of the clinic visits, followed by neighboring states of Kentucky (N = 119) and Alabama (N = 18). Figure 1 compares all outpatient clinic visits from April 2019 to all outpatient clinic visits in April 2020 based on the number of visits per county in Tennessee and the surrounding states. Davidson County, which is where Vanderbilt Children’s Hospital is located, had the most outpatient clinic visits in both months, so it is the densest on the maps.

**Figure 2.**
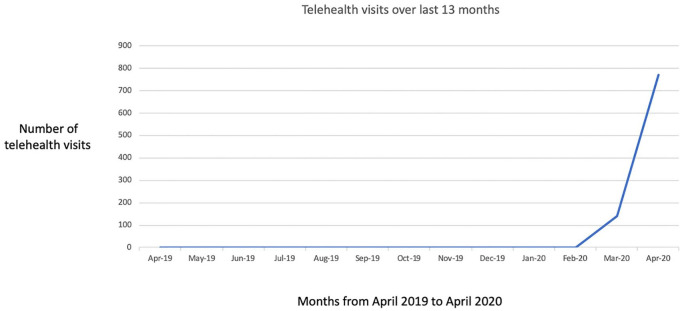
Telehealth visits for our Pediatric Otolaryngology Division over 13 months.

Over the past 13 months, our pediatric otolaryngology group has conducted a total of 912 telehealth outpatient visits, with 910 of those visits occurred during the months of March and April 2020. One telehealth visit was conducted in the months of October and November 2019 respectively. Figure 2 demonstrates this drastic rise in telehealth visits.

**Figure 3.**
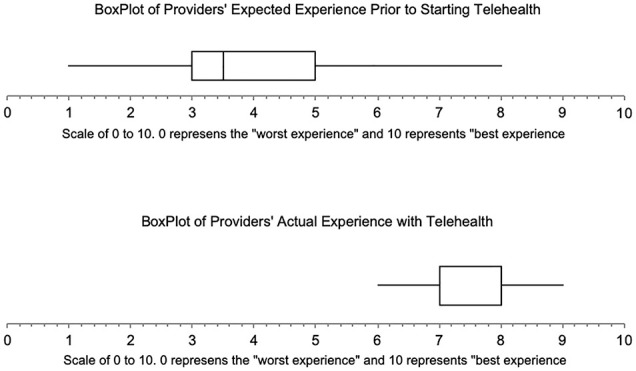
Boxplots comparing our provider’s anticipated experience of telehealth vs. their actual experience based on their Likert scale answers.

A total of 16 surveys were sent via email to all of our pediatric otolaryngology outpatient clinic providers and all 16 were completed and returned within 1 week. Only 2 of our 16 (12.5%**)** providers had performed a telehealth visit before the study period. Prior to the transition to telehealth, 11/16 (68.8%) providers felt telehealth would likely not be a viable platform to adequately care for our patients, despite 11/16 (68.8%) with previous experience with a teleconferencing platform such as Zoom. The primary concern was the limited physical exam with 11/16 (68.8%); 3/16 (18.8%) thought there would be difficulty with parent/patients not tolerating telehealth. On a scale of 0 to 10, with 10 being the “best experience” and 0 “worst experience,” providers rated their expected experience with telehealth prior to the start of our transition with a mean score of 3.94, 95% CI [3.0632, 4.8118]. They then rated their actual experience with telehealth after their March and April experience with a mean score of 7.5, 95% CI [7.113, 7.887], which was a statistically significant difference *P* < .00002. Figure 3 is a box plot of this data.

The majority of providers (10/16) reported 1 to 3 visits to become comfortable with the technology and billing aspect of the visit; 4/16 took only 4 to 8 visits and 2/16 took 9 to 15 visits to become comfortable with the new format. Most providers conducted their visits from home (13/16), while 3/16 preferred completing their telehealth visits at the hospital or office.

Zoom is the videoconferencing platform integrated with our EMR and that was used for the majority of telehealth with video visits. In the event of technical or connectivity issue, 6/16 utilized to Doximity (San Francisco, CA) application’s video conference capabilities, and 1 provider utilized Facetime (Apple, Cupertino, CA) on their smartphone. As the pandemic progressed and telehealth became entrenched, institutional research concluded that the Zoom platform provided the highest level of HIPAA security, and other platforms were banned. Provider behavior followed suit.

Providers were asked in a multiple-choice format about the best aspect of their telehealth experience, 8/16 felt it was the improved patient access to the care they needed, 4/16 thought it was the ability for patients and providers to avoid COVID-19 exposure, 2/16 felt it was the improved relationships and time with the patient families and the remaining 2/16 felt it was the flexibility in being able to care for patients in the comfort of their home.

Physical examination was felt to be limited. Only 2/16 were able to visualize the oropharynx/tonsils adequately over 50% of the time during their telehealth appointment, 6/16 between visualized it between 25% and 50% of the time, and 8/14 visualized the oropharynx/tonsils less than 25% of the time. No providers (0/16) were able to see the tympanic membrane (TM) via parents having otoscopes or ability to take pictures of the TM with other devices.

Providers were asked, following a full month experience, the likelihood that telehealth would be a part of their clinical practice in the future using a 0 to 10 scale (0 representing no role for telehealth, and 10 representing a fully integrated clinic with telehealth) when social distancing is eased and COVID-19 is not considered a major medical threat (mean at 7.56, range 2-10, standard deviation ±1.9).

Table 2 provides the qualitative survey questions and selected answers. The providers were asked which diagnoses were the most difficult to triage over telehealth. The most common answers were ear-related complaints and breathing concerns, such as noisy breathing or stridor. The reasoning provided for these difficulties is lack of physical exam details with no otoscope, no ability to peer into the ear canal, no audiologic evaluation, and no ability to perform flexible nasolaryngoscopy. When asked about perceived issues with telehealth from the patient’s perspective, almost universally the connectivity and ability to navigate the technology was mentioned by all providers, which led to frustration for the families.

**Table 2.** Selected Answers for Qualitative Questions From Survey Distributed to Our Pediatric Otolaryngology Division Outpatient Clinic Providers.

Q: What advice would you give to a pediatric otolaryngology provider using telehealth? Any physical exam caveats?
“Having 2 adults present for the visit helped. The best tonsil exam I got was when another adult shined a light into the patient’s mouth and the mom held the phone camera-we got a great picture. (I know one person could potentially do this with their phone, but some may have a hard time getting the light on their phone and still being active in the zoom meeting).”
“Encourage parents with children with ear tubes to get a digital otoscope from Amazon. You can use a spoon as a tongue depressor.”
“Having the child “make a pig nose” to try to do anterior rhinoscopy. I found it helpful to have the parents have another flashlight available to shine into the oropharynx to try to improve that part of the examination.”
“Give it a chance. Think outside the box for how you get the information you need. Question long standing thoughts on what is “required” to make a clinical decision.”
Q: What was the biggest obstacle with using telehealth perceived from the parent/patient perspective?
“Getting logged on and used to the platform. We do multiple visits per day. For the families it has been their first time using the platform”
“Not understanding all the steps to login, particularly how to start the visit even if they had MHAV set up correctly and had also downloaded Zoom.”
“Learning the new technology. Having the child present and able to cooperate with the camera. Many times this would be one parent trying to get a camera exam on a very mobile small child.”
“Accessing the visit due to internet issues, inability to understand instructions, forgetting time of appointment & not at home during telemedicine appt, not realizing child needed to be present

## Discussion

The first known patient in the United States with COVID-19 was diagnosed in Washington State on January 19, 2020. This was followed by the first reported death due to COVID-19 in the United States on February 29, 2020.^5^ By March 11, 2020 the World Health Organization declared COVID-19 as a pandemic,^6^ and shortly after that The White House and Centers for Disease Control (CDC) implemented a “30 days to slow the spread” plan for the United States that recommended avoiding social gathering of more than 10 people.^7^ This rapid timeline left hospitals and healthcare practices scrambling to not only provide necessary patient care with proper social distancing, but to also maintain current and future clinical and financial viability.^8^ This was the impetus for our transition to telehealth to meet those needs.

Recognizing that physicians across the United States were transitioning to telehealth and not being able to rely solely on face-to-face billing, the Centers for Medicare & Medicaid Services (CMS) broadened access to Medicare telehealth services and released guidance on this on March 30, 2020.^8,9^ CMS issued an 1135 waiver, which allows Medicare to pay for office, hospital, and other visits completed via telehealth retroactive to March 1, 2020. This waiver allowed for many changes to the ability for all providers, including pediatric otolaryngologists, to bill for these telehealth encounters. Prior to March 1st, 2020 Medicare required patients must live in a designated rural or underserved area, but now they may be anywhere in the country. State medical licensing requires a provider to have a valid license in the state in which the patient is located in order to use telehealth. As social isolation was expanded, the neighboring states of Kentucky, Alabama, and Mississippi very quickly granted temporary privileges allowing the use of telehealth across state boundaries. Additionally, prior restrictions requiring a HIPAA compliant telehealth platform were lifted, allowing platforms such as Apple FaceTime, Zoom, Google Hangouts, Skype, Zoom, to be utilized. There are several other changes under this waiver, but all of them are designed to improve access for patients and providers as well as ensure adequate reimbursement.^9^ Many of these telehealth visits performed are using real-time audio and video and billed using CPT codes 99201 – 99215.^8,9^ This new Medicare waiver recommends using modifier 95 (synchronous telemedicine service rendered via a real-time interactive audio and video telecommunications system) when previously (before March 1^st^, 2020) this was not required.^8,9^ Medicare has stopped requiring and using the modifiers GT (via interactive audio and video telecommunication systems) and CR (catastrophe/disaster related) but many of the private payers continue to use them.^8^

Prior to the start of the pandemic and outpatient clinic transition, only 12.5% (2/16) of our providers had ever used telehealth for a patient encounter. Given the lack of experience, it is not surprising that 68.8% (11/16) of our providers were skeptical or concerned about telehealth being a viable way to care for our patients. However, the actual experience of telehealth visits was significantly better (*P* < .00002) than the expected experience of telehealth. Philips et al.^10^ looked at cost savings with a telehealth program in a general otolaryngology clinic and found that there may be a decreased reimbursement rate for the otolaryngologist, but the value of convenience, decreased travel time, and increased efficiency was significant. Work efficiency may be increased during telehealth visits due to time between telehealth consults for academic or administrative purposes.^10^

Our EMR has the Zoom video conference application integrated into scheduled telehealth visits, which allows for the provider and patient to connect through the EMR. If there were technical or connectivity issues with this platform, 37.5% (6/16) were able to use the Doximity app with video connection successfully. Other options for video conferencing are available and our use of Doximity is most likely a result of our weekday morning faculty internal discussions. Each morning during this rapid transition our group met through video conferencing to troubleshoot clinical or surgical issues and improve our telehealth utilization. The Doximity application can be downloaded on a smartphone, and 1 advantage that it possesses is that it can customize the CallerID that your patients see and your cell phone number is never revealed when calling.

Of the 769 telehealth visits conducted in the month of April, 2020 32% (N = 246) were performed using telephone only. While these telephone only visits were an option in April 2019, they simply were not utilized. Patients or a patient’s caregiver must be enrolled in the institution’s MHAV online portal to set up a telemedicine visits with video. In order to sign up for MHAV the signup and activation instructions are sent electronically, so the parents or caregivers have to have an active e-mail to participate. For families that don’t have an e-mail or do not check their e-mail regularly, this inevitably puts them at a disadvantage of signing up for MHAV and subsequently scheduling telemedicine with video appointment, which may have limited our ability to have more telehealth visits with video. If there were technical or connectivity issues with the video conferencing, the visit would be converted to a telephone visit and billed accordingly. There is a “digital divide” in adoption of the telehealth technology as 1 study on pediatric surgery patients showed that barriers to telehealth include Hispanic race, non-English speakers, no post-secondary education, and uninsured status.^11^ This “digital divide” may have played a part in all of our telehealth visits that required interpretive services, as none of them were able to be completed via telemedicine with video despite an interpretive service available through our videoconferencing platform. All of the visits requiring interpretive services were completed via telephone only, which limits the physical exam aspect even further compared to face-to-face and telemedicine with video. We also saw a decrease in the percentage of patients (9.3%-7.5%) that required interpretative services when our practice switched to telemedicine services during the month of April 2020 compared to April 2019. As telemedicine programs are being developed and administered across the country, it is important to be aware of this and take steps to prevent it from happening, such as ensuring interpretive services are available for your telemedicine platform or being inclusive with other languages in marketing materials for telemedicine services. Despite widespread use of telehealth during the current pandemic there will likely continue to be a subset of the patient population that will not prefer telehealth as 1 survey reported there continues to be a desire to see a physician in person.^11^

There was a shallow learning curve for this new platform among our providers. The majority of our group (62.5% (10/16)) took only 1 to 3 visits to feel comfortable with all aspects of telehealth. The visit intake was felt to be a source of increased burden on providers during the transition from face-to-face visits to primarily telehealth visits. Typically, the clinical intake information is documented in the EMR by a medical assistant (MA) or licensed practical nurse (LPN) for face-to-face visits. When this transition to telehealth started, the onus of documentation in the EMR of chief complaint, extensive family history, social history, medical history, surgical history, allergies, and medications fell solely on the provider. MA’s and LPN’s in our clinic were largely reassigned to other roles and a full complement of assistants were not available to assist with intake. It was felt by the majority of providers that full responsibility for connectivity and intake would severely limit the efficiency and volume of visits that could be conducted via telehealth. Studies in telemedicine have shown that successful telemedicine programs minimize the burden for the healthcare providers, have programs that integrate well within the existing systems and have user-friendly technology.^12^ Over time a process was implemented in which a patient service representative would initiate clinic intake followed by a MA or LPN, who would establish telehealth video connection with the family and complete the clinic intake process all on the same video connection. They would have the ability to log in, and then log out, all while the patient was able to stay in the video conference for the visit. Once the patient was ready to be seen, the physician, NP, or PA would log into the visit either from their office on site at the hospital or their home. The providers felt that this system improved efficiency of the telehealth encounter and improved provider satisfaction.

Telehealth was not only a new endeavor for our staff and providers, but it was also a new concept for our patients. Telehealth in pediatric otolaryngology practices has been shown to result in cost savings, convenience for the families, and overall satisfaction for both parties. However, there are limitations with the physical exam.^13-15^ There is a much higher responsibility on the parents/caregivers to aid in the physical exam in telehealth, whereas this burden largely falls on the healthcare provider in face-to-face visits. 0% of our group was able to visualize a tympanic membrane during our 769 telehealth visits and only 12.5% (2/16) visualized the oropharynx and tonsils over 50% of the time. These low numbers are likely a result of our group’s lack of experience with telehealth and the quick transition to these type of encounters without much preparation. Without visualizing the tympanic membrane or oropharynx, it was difficult to make certain surgical or medical decisions regarding the patients and often necessitated recommending a face-to-face visit. Rapid advancements in technology and decreasing cost of technologies could allow for otoscopy to be performed in the home setting utilizing tele-otoscopy. There are several affordable tele-otoscopy options available to purchase in locations such as Amazon (Bellevue, Washington). Shah et al.^13^ looked at the ability of parents to use this type of technology to obtain images of their children’s tympanic membrane for clinical decision making. Despite watching the tele-otoscopy’s tutorial and parent’s self-reported comfort using the device, the images obtained by a parent were often not adequate for the consulting physician to make a correct diagnosis.^13^ Image quality was not the primary problem, rather the inability of the parental user to visualize the TM properly.^13^ This finding is not surprising considering even medical students and otolaryngology residents need repeat practice to reliably visualize the tympanic membrane. Motivated parents, particularly parents with children with frequent ear infections, eustachian tube dysfunction, lack of access to nearby providers, or other chronic otologic issues, could likely be trained to correctly visualize the tympanic membrane using tele-otoscopy technology. This would further increase the utility of telehealth and expand its capabilities. As our practice further refines its telehealth capabilities, there will be an increased emphasis and instruction to the parents prior to the visit of their role in aiding in the physical exam. These instructions will include guides to purchasing tele-otoscopy technologies and tutorial videos for best practices for physical exams and appropriate lighting. We expect telehealth visits to be integrated as part of the “new normal” for almost all medical practices as the COVID-19 pandemic continues to re-shape healthcare around the US and world. Table 2 provides other anecdotal experience and advice for physical exam caveats.

There were a few limitations to our study. First, the survey was not validated prior to implementation, so there may be recency bias or other biases that influenced the answers. This article was designed to give a provider’s experience on the telemedicine transition during the COVID pandemic, so another limitation is that patient’s experiences and possible survey results were not gathered. We also did not track or obtain objective data on the time for each visit and the efficiency, which may be useful in the future for understanding how to build templates and block time for these clinic appointments.

## Conclusion

Telehealth has become an integral part of our clinical practice as well as others around the US. Despite initial concerns over the utility of telehealth, currently all of our providers see telehealth as a continued part of their clinical practice post COVID pandemic. There are challenges with physical exam, particularly otoscopy and oropharyngeal exams, but with adequate information and preparation some of these obstacles could be overcome. As home physical exam technologies continue to expand, the utility of telehealth will likely continue to expand as well.
